# Single-incision laparoscopic surgery in gynecologic surgery: a single-institutional experience from Saudi Arabia

**DOI:** 10.12688/f1000research.12545.1

**Published:** 2017-09-07

**Authors:** Kareemah Salamah, Mohammed Abuzaid, Ahmed Abu-Zaid

**Affiliations:** 1 Department of Obstetrics and Gynecology, Women’s Specialized Hospital, King Fahad Medical City, Riyadh, Saudi Arabia; 2 College of Graduate Health Sciences, University of Tennessee Health Science Center, Memphis, Tennessee, USA; 3 College of Medicine, Alfaisal University, Riyadh, Saudi Arabia

**Keywords:** Laparoscopic single-site surgery; single-incision laparoscopic surgery; minimally invasive surgery; Gynecologic oncology; Saudi Arabia

## Abstract

**Background**: Laparoscopy is rapidly replacing laparotomy in the field of gynecologic surgery. Generally, there are limited data concerning the utility of single-incision laparoscopic surgery (SILS) in gynecologic surgery. Specifically, in Saudi Arabia, a third-world country, data are further limited; only one related study has been conducted so far. The purpose of this study is to retrospectively report our single-institutional experience of SILS in terms of feasibility, safety and perioperative outcomes in the management of various gynecologic conditions.

**Methods: **The study took place at the Women’s Specialized Hospital, King Fahad Medical City, Riyadh, Saudi Arabia. From January 2012 to May 2016, all gynecologic patients who underwent SILS procedures were analyzed for pre-, intra- and post-operative details. SILS was performed using a single multi-port trocar and standard laparoscopic instruments.

**Results**: A total of 54 patients underwent 66 SILS procedures. The median age and body mass index (BMI) were 36 years and 28.2 kg/m ^2^, respectively. Fourteen patients (26%) had ≥ 1 previous abdominal and/or pelvic surgeries. Twenty-four patients (44.4%) were nulliparous. The three most commonly performed SILS procedures were unilateral salpingo-oophorectomy (45.5%) and unilateral ovarian cystectomy (27.3%) and adhesiolysis (6.1%). The median operative time, estimated blood loss and hospital stay were 74 min, 50 ml and 1 day, respectively. Three patients required conversion to laparotomy, as follows: unidentified non-stopping bleeding source (n=1) and endometriosis stage IV resulting in difficult dissection (n=2). One patient developed post-operative incisional hernia that was treated surgically. The median patients’ post-operative pain (according to Wong-Baker FACES Foundation pain rating scale) within 4 hours was 2. At 4-week post-operatively, the median wound scar length (measured at outpatient clinic) was 2 cm.

**Conclusions**: SILS is feasible, safe and associated with acceptable clinical and surgical outcomes.

## Introduction

Minimally invasive surgery (laparoscopy) is rapidly replacing laparotomy in the field of gynecologic surgery ^1^. As opposed to laparotomy, laparoscopy provides plentiful benefits. Such benefits comprise: reduced postoperative pain, faster recovery to previous performance status, shorter hospital stay, better cosmesis, lower cost, reduced morbidity/mortality and overall improved surgical outcomes ^2– 4^.

One of the most notable advances in laparoscopy is the introduction of single-incision laparoscopic surgery (SILS). In contrast to the conventional laparoscopy that is regularly executed by using a total of three to five small incisions (5–20 mm each), SILS is performed by using a single small incision of the umbilicus to completely accomplish the laparoscopic surgical procedures ^1^. SILS has been demonstrated in retrospective and prospective studies to be feasible, safe and reproducible in managing various gynecologic conditions ranging from simple procedures (for example, adnexectomy) ^1, 3, 5– 9^ to highly complicated ones (for example, hysterectomy, complex pelvic masses and lymphadenectomy) ^10– 20^.

Generally, there are limited data concerning the utility of SILS in gynecologic surgery ^21^. Specifically, in Saudi Arabia, a third-world country, data are further limited; only one study about SILS in gynecologic surgery has been conducted so far ^22^.

The purpose of this study is to retrospectively report our single-institutional experience of SILS (feasibility, safety, and clinical/surgical outcomes) in the management of various gynecologic conditions.

## Methods

### Ethical approval

The study protocol was approved by the Institutional Review Board (IRB) of King Fahad Medical City [ID: 15-477].

### Setting and design

This was a retrospective study from January 2012 to May 2016 which took place at the Women’s Specialized Hospital, King Fahad Medical City, Riyadh, Saudi Arabia — a tertiary healthcare institution. At our institution, as of January 2012, SILS has been the standard management option for gynecologic patients who met the inclusion criteria, namely: age less than 90 years, body mass index (BMI) less than 50.0 kg/m ^2^, acceptable preoperative performance status and laboratory profile, technically resectable early stage adnexal or endometrial lesions, and signed written informed consent by patients after being well-informed about the risks/benefits of SILS.

### Patients

From January 2012 to May 2016, the medical records of all gynecologic patients who underwent SILS procedures for gynecologic conditions were retrospectively analyzed for pre-, intra- and post-operative details. Pre-operative details comprised: age, BMI, previous abdominal and/or pelvic surgeries, concomitant co-morbidities and parity. Intra-operative details comprised: type of procedures performed, conversion to conventional laparotomy, operative time (OT), estimated blood loss (EBL), lesion size and intra-operative morbidity/mortality. OT was defined as the interval from the initial umbilical skin incision to its closure. Post-operative details comprised: lesion pathology, patients’ self-reported recommendation (yes/no) of SILS to others, hospital stay, and post-SILS wound size, morbidity, mortality and pain. The post-SILS wound size was measured at 4 weeks at the outpatient clinic. Post-SILS morbidity/mortality were defined as any surgery-related complications/death up to 24 weeks postoperatively. The self-reported scores for post-operative pain were documented within 4 hours postoperatively by the operating surgeon/ward nurse using the Wong-Baker FACES Foundation pain rating scale.

### Surgical technique

All SILS procedures were primarily performed by a single surgeon from Section of Gynecologic Oncology, Department of Obstetrics and Gynecology. Procedures were performed under general anesthesia. Patients were placed in supine/lithotomy position, prepped and draped according to the hospital protocol. A 15 mm transverse intra-umbilical incision was made and skin edges were grabbed with Allis clamps. Afterwards blunt dissection was performed to create a 15 mm opening into the peritoneum. Then, Medtronic SILS ^TM^ device (Medtronic, Minnesota, USA) was introduced into the peritoneal cavity using packing forceps. The device has one gas inlet and 3-access ports (two 5-mm ports and one 12-mm port). Then, pneumoperitoneum was accomplished using carbon dioxide (CO _2_) through the gas inlet valve. Various laparoscopic instruments (rigid 0-degree and prebent) were used as deemed appropriate by the operating surgeon to avoid instrumental clashing and improve the operation field. The resected specimens were removed using the 12-mm port and endobag to allow for intact removal of the surgical specimen. The rectus sheath was closed with number 0 maxon sutures and skin closed with 3-0 vicryl sutures. In case of failure of SILS, the procedure was converted to either conventional laparoscopy or laparotomy.

### Follow-up

All patients were followed up for at least 6 months post-operatively at the outpatient clinic.

### Analysis

The descriptive raw data are reported in Dataset 1. Data were analyzed with Microsoft Excel 2013. Whenever possible, data were presented as percentages, median ± standard deviation (SD) and range values.

Descriptive Raw Data Pre-operative, intra-operative and post-operative details Click here for additional data file. Copyright: © 2017 Salamah K et al. 2017 Data associated with the article are available under the terms of the Creative Commons Zero "No rights reserved" data waiver (CC0 1.0 Public domain dedication).

## Results

A total of 54 patients underwent SILS procedures. Characteristics of the patients are depicted in Table 1. The median age ± SD was 36 ± 16.9 years (range: 15–88) whereas the median BMI ± SD was 28.2 ± 6.1 kg/m ^2^ (range: 18.9–44.1). A total of 14 patients (26%) had ≥ 1 previous abdominal and/or pelvic surgeries. Regarding parity, 24 (44%), 2 (4%) and 28 (52%) patients were nulliparous (para 0), primiparous (para 1) and multiparous (para 2+), respectively.

**Table 1.** Characteristics of SILS patients (pre-operative details).

Patients (n=54)	
Median age ± standard deviation, (range)	36 ± 16.9 years (15–88)
Median body mass index ± standard deviation, (range)	28.2 ± 6.1 kg/m ^2^ (18.9–44.1)
Previous abdominal and/or pelvic surgeries None 1 surgery 2 surgeries 3 surgeries	40 (74.1%) 8 (14.8%) 5 (9.3%) 1 (1.9%)
Type of surgery Laparoscopy Laparotomy	3 (14.3%) 18 (85.7%)
Concomitant co-morbidities None 1 2 3 and more	32 (59.3%) 10 (18.5%) 8 (14.8%) 4 (7.4%)
Parity None (para 0) 1 (para 1) 2 and more (para 2+)	24 (44.4%) 2 (3.7%) 28 (51.9%)

The intra-operative details of SILS are portrayed in Table 2. A sum of 66 SILS procedures were carried out. A total of three patients required conversion to laparotomy. The three most frequently performed SILS procedures were unilateral ovarian salpingo-oophorectomy (45.5%), unilateral ovarian cystectomy (n=27.3%) and adhesiolysis (n=6.1%). The median size of resected lesions ± SD was 12 ± 8.6 cm (range: 1.3–50). The median OT and EBL ± SD were 74 ± 39.4 min (range: 40–200) and 50 ± 271.7 ml (range: 20–2000), respectively. None of the patients experienced intra-operative death.

**Table 2.** Intra-operative details of SILS procedures.

Patients (n=54), Procedures (n=66)	
Total procedures performed Unilateral salpingo-oophorectomy Bilateral salpingo-oophorectomy Unilateral ovarian cystectomy Bilateral ovarian cystectomy De-torsion Adhesiolysis Contralateral ovarian cyst aspiration Laparoscopic-assisted vaginal hysterectomy Total laparoscopic hysterectomy	30 (45.5%) 3 (4.5%) 18 (27.3%) 2 (3%) 1 (1.5%) 4 (6.1%) 3 (4.5%) 3 (4.5%) 3 (4.5%)
Intra-operative conversion to laparotomy	3 (5.6%)
Intra-operative mortality	0
Median size (greatest dimension) of resected lesions ± SD (range)	12 ± 8.6 cm (1.3–50)
Median operative time ± SD, (range)	74 ± 39.4 min (40–200)
Estimated blood loss ± SD, (range)	50 ± 271.7 ml (20–2000)

The post-operative details of SILS are shown in Table 3. There were 44 (81.5%) and 10 (18.5%) benign and malignant lesions, respectively. The median patients’ self-reported scores for postoperative pain was 2 ± 1.5 (range: 0–6). The median hospital stay was 1 ± 0.7 days (range: 1–4). At 4-week post-operatively, the median length of the wound scar (measured at the outpatient clinic) was 2 ± 0.4 cm (range: 1.5–2.5). During 6-month follow-up, one patient developed postoperative complication (incisional hernia) that was managed surgically.

**Table 3.** Post-operative details of SILS procedures.

Lesion pathology Benign Malignant	44 (81.5%) 10 (18.5%)
Median hospital stay ± standard deviation, (range)	1 ± 0.7 days (1–4)
4-week post-operative wound size ± standard deviation, (range)	2 ± 0.4 cm (1.5–2.5)
6-month major post-operative complications	1 (1.8%)
6-month SILS-related mortality	0
Median patients’ post-operative pain ± SD, (range) *	2 ± 1.5 (0–6)
Patients’ recommendation of SILS to others	53 (98.1%)

*Wong-Baker FACES Foundation pain rating scale

## Discussion

SILS is one of the cutting-edge developments in the field of minimally invasive surgery ^1^. However, there are insufficient data concerning the feasibility, safety and perioperative outcomes of SILS in gynecologic surgery in Saudi Arabia.

To the best of our knowledge, this is the second ever study in Saudi Arabia to report the single-institutional experience of SILS in gynecologic surgery. Moreover, it is among the first studies originating from third-world countries that examined the efficacy of SILS in gynecologic surgery. Our study demonstrated that SILS is feasible, safe and associated with acceptable clinical and surgical outcomes. Nearly all patients (98.1%) agreed to recommend SILS to other patients.

The only study of SILS in Saudi Arabia was reported by Al-Badawi *et al.* ^22^. They reported their single-center experience of SILS in the management of benign salpingo-ovarian pathologies. They had 80 patients and a total of 104 performed procedures. They concluded that SILS in the management of benign salpingo-ovarian conditions was generally feasible, potentially safe, and associated with satisfactory operative and postoperative outcomes. As opposed to the Al-Badawi *et al.* study that included only adnexectomy procedures ^22^, our study included far more complex (non-adnexectomy) procedures, such as: SILS laparoscopic-assisted vaginal hysterectomy (LAVH, n=3), and SILS total laparoscopic hysterectomy (TLH, n=3). Several studies showed that SILS approach can be used to successfully perform both TLH and LAVH procedures ^10– 20^.

In our study, 3 patients required conversion to laparotomy for several reasons: unidentified non-stopping bleeding source (n=1) and endometriosis stage IV resulting in difficult dissection (n=2). Patient safety should never be recklessly put in danger at any given time. Therefore, conversion from SILS to laparotomy —whenever at the best interests of patients— is more significant than mere cosmetic concerns. Also, such conversion from SILS to laparotomy should not be prematurely judged as a surgical failure or an incompetence of the operating surgeon to perform SILS procedures.

Patients with high BMI are regarded as a challenging group for SILS procedures due to the anticipated high intraperitoneal fat contents which can cause port access difficulties and garble the field visualization ^23^. It has been recommended that patients with BMI less than 28 kg/m ^2^ are regarded as suitable patients for SILS ^24^. In our study, more than 50% of patients had BMI of more than 28 kg/m ^2^ and none experienced eventful surgical courses.

Moreover, patients with previous abdominal and/or pelvic surgeries are regarded as a challenging group for SILS procedures due to the anticipated dense surgery-related adhesions and difficult dissection ^23^. In our study, around 11.1% of patients had 2 or more previous surgeries; however, none of them required conversion to laparotomy.

Furthermore, port-site metastasis in malignant gynecologic oncology is sometimes worrisome to both patients and laparoscopists. However, the frequency of port-site metastasis following laparoscopic procedures in women with malignant gynecologic pathology is very low (less than 1%) ^25^. Numerous studies showed feasibility, safety and reproducibility of the SILS for management of precancerous pathologies and select early-stage ovarian and uterine malignancies ^10, 11, 13^. Thus, it can be concluded that SILS should not be avoided in the management of patients with early-stage gynecologic malignancy. In our study, 10 patients had malignant pathologies and successfully underwent SILS procedures without proof of port-site metastasis at a median follow up of 29 months (range: 23–37).

In brief, apposite selection of patients for SILS is of great significance. According to our study, our recommendations for patients who may be eligible candidates for SILS procedures comprise: BMI less than 40 kg/m ^2^, less than 3 previous surgeries (irrespective of laparoscopic and/or laparotomic), presence of native umbilicus and early-stage gynecologic malignancy.

SILS is not without its technical challenges which are well-documented in literature. Such challenges generally comprise: the limited triangulation and retraction capacities as well as the camera/laparoscopic instruments conflict that can distort proper surgical field exposure ^26^. Measures to prevail these downsides are continuously in progress and comprise the introduction of flexible and prebent laparoscopic instruments of different lengths ^22^.

Similar to conventional laparoscopy, SILS is a surgical procedure that necessitates a great deal of fine-motor hand dexterity. Two studies documented that around 10 to 15 procedures were required to perform SILS adroitly which was reflected on reduced operating time ^10, 27^. Many measures have been advocated to improve the learning curve, for example: watching live/recorded procedures, virtual simulation and hands-on practice on animals ^26, 28^. There are serious ongoing efforts to implement the above-mentioned measures at our institution.

Our future research directions include: 1) the learning curve of SILS at our institution, and 2) comparison between SILS and conventional laparoscopy with regard to management of various gynecologic pathologies.

Our study has several limitations and comprise: retrospective study design, lack of control group (conventional laparoscopic), relatively small sample size, single-institutional experience and patients subjective self-reported scores for postoperative pain.

## Conclusion

This is the second single-institutional experience of SILS in gynecologic surgery from Saudi Arabia. Our study demonstrated that SILS is feasible, safe and associated with acceptable clinical and surgical outcomes. SILS is an operator-dependent procedure and requires advanced surgical training.

## Data availability

The data referenced by this article are under copyright with the following copyright statement: Copyright: © 2017 Salamah K et al.

Data associated with the article are available under the terms of the Creative Commons Zero "No rights reserved" data waiver (CC0 1.0 Public domain dedication). 

Descriptive Raw Data- Pre-operative, intra-operative and post-operative details

10.5256/f1000research.12545.d176453 ^29^
