# Supplementary material for: Single-incision laparoscopic surgery in gynecologic surgery: a single-institutional experience from Saudi Arabia
Source: F1000Res. 2017 Sep 7;6:1657. [Version 1] doi: 10.12688/f1000research.12545.1 (PMC5609086; doi:10.12688/f1000research.12545.1)
Supplement: Peer review history

# Peer review: Referee response for version 1

**DOI:** 10.5256/f1000research.13584.r25796

**Published:** 2017-09-21

**Authors:** Sarfraz Ahmad

**Affiliations:** 1 Florida Hospital Medical Center, Orlando, FL, USA

With great interest I read the paper by Salamah *et al.* The authors reported their single-center experience of single-incision laparoscopic surgery (SILS) in the field of gynecologic surgery from a tertiary healthcare center in Saudi Arabia. The topic is of great significance since this study is only the second ever from Saudi Arabia, and will add valuable data to the limited regional peer-reviewed literature.

**Introduction: **The authors introduced the topic appropriately, defined terminology of SILS, and highlighted the significance of the study thereby clearly mentioned the objectives of study.

**Methods:** The ethical approval for the study was obtained. Appropriate descriptions of the study design/setting, research subjects and surgical procedure are provided. The study was descriptive, retrospective, and one-group of patients. Only simple statistical tests were carried out, i.e., calculations of numbers/percentages, SDs and ranges.

**Results: **Overall, the results section reads well reporting the major findings. Results were presented in simple tables and divided into pre-, intra- and post-operative categories. There are some repetitions between the text and the tables which makes easier to understand the findings. In Table 2, perhaps the authors should correct the “Type of Surgery” data: i.e., Laparoscopy=51, Laparotomy=3 (instead of 3 and 18, respectively) and appropriate percentages.

**Discussion:** It is succinct and properly reflecting on the study’s main findings without unnecessary details. The significance of study was re-highlighted. Brief comparisons with the the existing peer-reviewed literature were made with regards to aspects pertaining to the pre-operative details such as BMI and previous surgery. Most importantly, the study findings were directly compared-and-contrasted to the only study from Saudi Arabia (by Al-Badawi *et al.* 2016) which is a plausible and appreciated move. Study’s future directions and limitations are adequately pinpointed.

**Conclusion:** Reads very well and reflects on the study’s main conclusions.

**Overall Comments:** The manuscript is very well-written in terms of English language, grammar, word counts, clarity, logical sequence, citations, and appropriate references cited. The strong points of paper are: 1) being the second in Saudi Arabia, which highlights the significance of the study to warrant additional peer-reviewed publication based on scientific soundness, despite no novelty; and 2) the study is of great importance in terms of regional interests as it enriches the scarce limited peer-reviewed literature on the topic. The study weakness mainly revolve around the methodology part as this is a mere descriptive retrospective study without a control-group and detailed statistical calculations, which makes it a “less” strong evidence; however, the study limitations are duly acknowledged by the authors. I highly recommend the authors to pursue future studies proposed in their manuscript, as these directions are going to have more impact and contribute substantially to the body of existing of peer-reviewed literature regionally and internationally. More strong-evidence-based study design should be considered too, such as prospective and controlled-study designs. A cost-effective study comparing conventional vs. SILS in gynecologic surgery indications may be an interesting topic, especially from a third-world country with relatively constrained healthcare economy.

**Final Remarks and Decision:** According to the scope/policy of the F1000 Research Open-Access journal, the study is “scientifically sound” and “suitable” irrespective of the perceived level of novelty. Therefore, I approve the study for publication in its current draft.

**Conflict of Interest:** I report no conflict of interest pertaining to this manuscript.

I have read this submission. I believe that I have an appropriate level of expertise to confirm that it is of an acceptable scientific standard.

## References

[1] SalamahKAbuzaidMAbu-ZaidA: Single-incision laparoscopic surgery in gynecologic surgery: a single-institutional experience from Saudi Arabia. *F1000Research*.2017;6: 10.12688/f1000research.12545.1 10.12688/f1000research.12545.1 28979769PMC5609086

# Peer review: Referee response for version 1

**DOI:** 10.5256/f1000research.13584.r25813

**Published:** 2017-09-18

**Authors:** Ahmed Nazer

**Affiliations:** 1 Department of Obstetrics and Gynecology, McGill University, Montreal, QC, Canada

Small descriptive study about SILS. Based on the presets data, it is difficult to comment about the safety of this approach for TLH or LAVH as the number is very small and there is no informations about the indications of the hysterectomies.

 Overall promising results and worth to publish .

I have read this submission. I believe that I have an appropriate level of expertise to confirm that it is of an acceptable scientific standard.
